# “Fighting an uphill battle”: experience with the HCV triple therapy: a qualitative thematic analysis

**DOI:** 10.1186/1471-2334-14-507

**Published:** 2014-09-18

**Authors:** Manuela Rasi, Patrizia Künzler-Heule, Patrick Schmid, David Semela, Philip Bruggmann, Jan Fehr, Susi Saxer, Dunja Nicca

**Affiliations:** Cantonal Hospital St. Gallen, Division of Infectious Diseases and Hospital Epidemiology, 9007 St. Gallen, Switzerland; Cantonal Hospital St. Gallen, Division of Gastroenterology/Hepatology, 9007 St. Gallen, Switzerland; Arud Centres for Addiction Medicine, 8005 Zürich, Switzerland; Division of Infectious Diseases and Hospital Epidemiology, University Hospital Zürich, 8006 Zürich, Switzerland; University of Zürich, 8006 Zürich, Switzerland; Institut of Applied Nursing IPW-FHS St. Gallen, University of Applied Science, 9000 St. Gallen, Switzerland; Institute of Nursing Science, University of Basel, 4056 Basel, Switzerland

**Keywords:** Treatment experience, Hepatitis C, Protease inhibitor, Self-management

## Abstract

**Background:**

Hepatitis C virus (HCV) infections are a severe burden on public health worldwide, causing mortality rates triple that of the general population. Since 2011, for both therapy-naive and therapy-experienced genotype 1 patients, the first generation of direct acting antivirals (DAAs), i.e., the protease-inhibitors (PI) telaprevir and boceprevir have been added to existing dual therapies. The therapeutic effect of the resulting triple therapy is striking; however, treatment regimens are complex and commonly cause side effects. Little is known of how patients implement therapy in their daily lives, or of how they deal with these effects.

This study aims to describe HCV patients' experiences with protease-inhibitor-based triple therapy and their support needs.

**Methods:**

A qualitative design was used. Patients from three outpatient clinics, with ongoing, completed or discontinued PI treatment experience were recruited using a maximum variation sampling approach. Open-ended interviews were conducted and analyzed using thematic analysis according to Braun & Clarke (Qual Res Psychol 3:77-101, 2006).

**Results:**

Thirteen patients participated in the interviews. All described themselves as highly motivated to undergo treatment, since they saw the new therapy as a “real chance” for a cure. However, all later described the therapy period as a struggle. The constitutive theme–“Fighting an uphill battle”– describes the common existential experience of and negative consequences of coping with side effects. The processes that fostered this common experience followed three sub-themes: “encountering surprises”, “dealing with disruption” and “reaching the limits of systems”.

**Conclusion:**

HCV patients undergoing outpatient protease-inhibitor-based triple therapy need systematic support in symptom management. This will require specially trained professionals to advise and support them and their families, and to provide rapid responses to their needs throughout this complex course of therapy. As the generation of DAAs for all genotypes, are expected to have less severe side effects, and many HCV patients require treatment, this knowledge can improve treatment support tremendously, especially for patients who are quite difficult to treat. Furthermore, these findings are helpful to illustrate development in HCV treatment.

**Electronic supplementary material:**

The online version of this article (doi:10.1186/1471-2334-14-507) contains supplementary material, which is available to authorized users.

## Background

Hepatitis C virus (HCV) infections are a severe burden on public health worldwide. As the main causes of cirrhosis and cancer of the liver, they are the most frequent indication for liver transplantation [1, 2]. It is estimated that more than 160 million people currently live with chronic HCV, including about 15 million in the WHO European region [3, 4]. In the United States, the HCV-related mortality rate is triple that of the general population [5], surpassing that of HIV [6].

Antiviral therapies' sustained virological response (SVR), indicating that the patient is cured, is defined as the absence of target virus RNA 24 weeks after the end of therapy [7]. For more than 10 years, the standard treatment for all six human-occurring HCV genotypes has been a combination of pegylated interferon (IFN) and ribavirin (RBV) (dual therapy), with SVR rates of 40-50% for genotype 1 (the most common genotype in the United States and Europe) [1, 8]. However, since 2011, the first-generation direct acting antivirals (DAAs), i.e., the protease-inhibitors (PI) telaprevir (TPV) and boceprevir (BOC), have been used in both therapy-naive and therapy-experienced genotype 1 patients. Adding one of these PIs to dual therapy (IFN/RBV) produces a "triple therapy" (IFN/RBV/PI), leading to SVR rates as high as 70% [8–10]. Treatment time of triple therapy is 24 – 48 weeks. TPV is prescribed at treatment start, while BOC is preceded by a 4-week lead-in phase with IFN/RBV. For monoinfected patients with an optimal virological response, treatment time can be shortened.

HCV care is usually performed in outpatient clinics. This presumes that patients who start treatment are capable of taking oral medication linked to dietary requirements, e.g., TPV along with 21 grams of fat, and self-administering subcutaneous injections at least twice daily. With triple therapy, in order to prevent resistance, the protease-inhibitor must be taken punctually three times a day, i.e., every 8 hours [11]. Moreover, some evidence suggests that a high proportion (>80%) of patients can achieve the same results regardless of whether they take TPV twice or three times daily [12]. However it has been documented that some patients who lack effective support systems, or whose medical, behavioral or social problems complicate treatment, do not receive access to treatment [13, 14].

Side effects of anti-HCV dual therapy can include flu-like symptoms, fatigue, depression, skin problems or changes in blood counts [15]. Under triple therapy, additional serious side effects may occur, such as pronounced anemia, diarrhea, dysgeusia, severe skin rash or anorectal events [16]. Side effects have shown to be a serious burden for patients and can even be life –threatening [17, 18].

The complexity of the treatment plan and dealing with various symptoms of the disease and its treatment places great self-management demands on the patient. By definition, self-management encompasses the day-to-day activities a chronically ill patient performs to minimize the negative consequences of his or her condition. Such actions vary based on the persons’ perceptions, experiences and expertise, but share the focus of optimizing health outcomes and improving overall wellness [19, 20]. During the course of HCV, patients have to deal with numerous self-management tasks, including: a) understanding the facts about HCV; b) making lifestyle changes to maximize liver health; c) coming to terms with their diagnosis, mortality and transmission probabilities d) dealing with disclosure, discrimination and stigma; e) organizing emotional support; f) managing the disease alongside work and family; and g) making ongoing treatment decisions [21–23]. Once a patient has made the decision to start treatment, the integration of dual or triple therapy into daily life and the management of symptoms become central self-management tasks with major impacts on health outcomes. For example, medication adherence defined by the World Health Organization (WHO) as “the extent to which a person’s behaviour – taking medication, following a diet, and/or executing lifestyle changes–corresponds with agreed recommendations from a health care provider” [24] during dual therapy is often insufficient, even though non-adherence has been linked to insufficient virological response [25, 26]. Studies have revealed that achieving an SVR via triple therapy is likewise adherence-dependent, i.e., 80% of BOC doses must be taken over the assigned treatment period [27]. However, a study of dual therapy showed e.g. 100% adherence in 38% of patients over the full initially intended treatment period [26].

Side effects are additional challenges and have been shown to negatively impact treatment adherence [28, 29]. Preliminary evidence has also indicated that interventions improve HCV patients’ adherence and thus increase the possibility of SVR [30–32]. However, to date, it is unclear which intervention content, structure or application is most effective. Across several chronic conditions such as HIV, arthritis and diabetes, an understanding of the patients’ illnesses and treatment experiences has strengthened the development of effective self-management interventions [33, 34].

So far, though, little is known about how patients implement triple therapy in their everyday lives and how they deal with side effects [35]. To enable the development of effective intervention programs, this study addresses the question of how HCV patients experience the everyday dealing with protease-inhibitor-based triple therapy and what support they need. As the generation of DAAs for all genotypes, including recently-introduced interferon-free regimes, are expected to have less severe side effects, and many HCV patients require treatment, this knowledge can improve treatment support tremendously. However, depending on the regimen, IFN and RBV are still recommend by the European Association for the Study of the Liver (EASL) and are always appropriate [36].

## Methods

A qualitative research approach was selected for the description of patients’ triple therapy experiences. This is appropriate when prior knowledge and empirical evidence are limited and understanding of patients’ perspectives is needed to develop culturally sensitive instruments and effective interventions [37]. Methodologically, the thematic analysis used for this inquiry is aligned with a constructivist orientation [38]. Based on this approach, we assume that individuals construct their own realities and meanings from their culturally available language and subjective experience to make sense of their daily lives. Meaning is understood as generated and co-created in researchers’ interactions with the data [39]. More concretely, we theoretically oriented the inductive identification of thematic patterns (themes) to reflect commonalities and differences in patients' experiences to produce clinically relevant knowledge [40].

### Study site/study group

The study was performed between October 2012 and February 2013 in three outpatient clinics: an infectious disease clinic and a gastroenterological/hepatological clinic, both at the same hospital, and a clinic for internal medicine and infectious diseases at a center for addiction medicine. This multicenter approach was chosen because few patients were being treated with triple therapy at the time of investigation. The aim was to recruit 10 – 15 patients–a number usually sufficient to provide meaningful insights into patient experiences through the identification of themes reflecting commonalities and differences. Inclusion criteria were: being 18 years of age or older, having four weeks or more experience with a protease-inhibitor-based HCV therapy (ongoing, completed or discontinued), being in outpatient medical care and having the ability to participate in an interview in German. Persons whose treating physicians judged the additional burden of study participation unacceptable in relation to their emotional state (e.g., pronounced depressive symptoms, suicidal ideation) were excluded. Recruitment used maximum variation sampling [41]. The principle of this sampling strategy is to interview as diverse a selection of people as possible. In a relatively small sample, as is usual in qualitative studies, this maximum variation sampling allows the integration of a broad range of experiences into data analysis. Considering that limited sampling information was available from clinical experience and literature, we sought to include subjects who, according to their characteristics and circumstances, would add different experiences to the analysis. To obtain the maximum variation sample we assured that participants differed according to gender, age group, route of HCV infection and PI in their treatment regimen (TPV or BOC). An additional effort was made to include persons with HIV/HCV-Coinfection and intravenous drug users (IDUs). As many members of this patient group need treatment but are known to face multiple additional medical and social challenges, it was deemed important to include their experiences [42].

Patients were informed about the study either during a medical check-up visit or by telephone by a healthcare provider whom they knew. Of the 15 persons contacted about the study, 13 agreed to participate. Of the reasons for refusal, one patient's travel plans conflicted with the study timetable and the other did not wish to spend the necessary time participating.

### Data collection

Data collection was performed via open-ended interviews. Prior to the study, guidelines were developed with open questions on experiences concerning illness, treatment, symptoms and interactions with significant others, including healthcare providers. For all participants the interview started with the following open question: “Would you please tell me about your day-to-day experience with hepatitis C treatment?” Based on the patient’s narratives, mentioned topics were explored in depth. The interviewer asked about any topics listed in the interview guide but not brought up by patients. Data analysis was started after the first three interviews.

Throughout the study, we continuously integrated and discussed themes from preliminary analysis to identify commonalities and differences between participants. Following Thorne's position that being able to explore all possible theoretical aspects of a phenomenon is useful in the discovery of basic social processes, but is less logical in documenting human health experience (where the focus is on cases' differences and commonalities) [40], we did not aim for theoretical data saturation.

The first author (MR), an experienced addiction medicine and infectious diseases nurse and Master’s student, conducted the interviews. She was supervised by the last author (DN), an experienced qualitative researcher. She did not provide nursing care for any of the participants.

The participants decided on the locations for their interviews. Four were conducted at home and nine in the hospital after a medical consultation. At one interview a 2-year-old child was present; at another the patient’s partner attended to fill in the gaps in her partner’s memory during a life-threatening episode. Otherwise, only the interviewer and the participant were present. The interviews lasted 30–64 minutes (mean: 45 minutes), and were recorded and transcribed verbatim, omitting the patient’s or any other names, as well as the place of interview. Transcripts were not returned to patients for comment.

After the open-ended interviews, sociodemographic and medical data were collected using a structured questionnaire (date of birth, gender, first diagnosed, former dual therapy experience, triple therapy status (e.g., ongoing, completed or discontinued), PI used, residence situation, occupation, ability to work). Field notes were also made after each interview.

### Data analysis

The transcripts were systematically analyzed according to the thematic analysis method described by Braun and Clarke [38]. This procedure enabled the researchers to identify significant recurring topics, interpret them and set them in relation to one another in a constituent pattern. Along with the analytic steps, which followed Braun and Clarke [38], shown in an additional file (see Additional file 1: Table S1), the role of each author in this process is presented in Table 1. The software program ATLAS.ti 7.0 (Scientific Software Development GmbH Berlin) was used to support data analysis. Following the checklist for good qualitative analysis (see Additional file 2: Table S2), each step of the analysis was discussed with researchers and HCV care experts who had not been directly involved in development of the themes used.

**Table 1:** **Six phases of thematic analysis by Braun & Clarke**[38] **and role of authors**

1. Familiarising yourself with your data	The accuracy of the transcribed interviews was checked. Transcripts were read by three authors (MR PK DN) and notes of initial ideas were made and discussed in the research team.
2. Generating initial codes	All transcripts were coded systematically using inductive methods. The first five interviews were coded by three authors (MR PK DN) and codes were discussed until consensus was reached. The following eight transcripts were coded by the first author (MR), codes and quotes were presented and discussed with PK DN. With the support of Atlas.ti codes remained associated with the transcripts (quotes).
3. Searching for themes	Based on the code lists the research team (MR PK DN) summarized several codes into meaningful themes whose relevance emerged across several interviews. A preliminary description of the main and subthemes was made.
4. Reviewing themes	The first author (MR) checked the preliminary description of themes with the original data (transcripts). Inconsistencies were discussed in the research group. The first author undertook adjustments and defined the main theme and subthemes. The last author (DN) who has expertise in thematic analysis reviewed these themes and together with the first author related themes to a thematic map.
5. Defining and naming themes	The first author (MR) returned to the transcripts and worked out the specific thematic content, then, with DN, worked out the overall story line. The preliminary results were presented to authors and experts in care of HCV patients. According to this discussion some refinements were made.
6. Producing the report	The first author (MR) wrote a first draft of the scientific report, and selected vivid quotes to illustrate themes. The last author (DN) reviewed the report and necessary adjustments were made. The report was submitted to the research team for critical assessment, and the team's responses were recorded.

After data analysis was finalized, results were translated into English by a professional translator. The translation was checked and adapted by the research team. Very specific difficult-to-translate wordings were added in parentheses in the language of origin.

The 15-item criteria checklist developed by Braun und Clarke [38] was used to guarantee the quality of data. An additional file shows this checklist (see Additional file 2: Table S2). All participants were provided detailed verbal and written information about the study, and all signed written informed consent forms. The study was approved by both relevant bodies of the Swiss Association of Ethics Committees for clinical trials in St.Gallen (EKSG, reference number 12/122 L/1B) and Zürich (KEK-ZH, reference number 2012–0484). To ensure an accurate and transparent manuscript reporting it adheres to RATS guidelines.

## Results

Of the eight men and five women who participated in the study (aged 36–63 years), eight were living with partners or family, three alone and two in an institution. Their HCV diagnoses had been made 1.5 – 28 years previously. Before starting triple therapy, twelve of thirteen patients had experienced fibrosis stages that indicated treatment. Eight had prior experience with dual therapy and five had none. For their triple therapy, the PI Telaprevir had been administered to nine persons and Boceprevir to four. Demographic and medical data are presented in Table 2.

**Table 2:** **Patients characteristics**

Variable		Patients (n = 13)
HCV Genotype 1		13 (100%)
Fibrosis stage 0-1		1 (8%)
Fibrosis stage 2		3 (23%)
Fibrosis stage 3		3 (23%)
Fibrosis stage 4		6 (46%)
Gender	Male	8 (62%)
	Female	5 (38%)
Mean age in years (range)		49 (36–63)
Transmission	IDU^1^	6 (46%)
	Blood products	2 (15%)
	Unsafe sex	1 (8%)
	Unsafe tattooing	1 (8%)
	Unknown	3 (23%)
Opioid substitution		3 (23%)
Multimorbidity^2^		4 (30%)
First diagnosed mean years (range)		12.5 (1.5 - 28)
Treatment experience	Naive	5 (38%)
	Dual therapy 1×	7 (54%)
	Dual therapy 2×	1 (8%)
Protease inhibitor	Telaprevir	9 (70%)
	Boceprevir	4 (30%)
Status proteaseinhibitor therapy	Ongoing	5 (38%)
	Completed	5 (38%)
	Discontinued^3^	3 (23%)
Residence situation	With Partner/Family	8 (62%)
	alone	3 (23%)
	Institution^4^	2 (15%)
Occupation before triple therapy		10 (80%)
Job related working ability during triple therapy	0-25%	5 (50%)
	26-50%	1 (10%)
	51-75%	1 (10%)
	76-100%	3 (30%)

^1^Intravenous drug user.

^2^One patient with HIV, haemophilia, Depression or Schizophrenia.

^3^Two patients due to neutropenia, one patient due to unbearable side effects.

^4^Mental hospital, assisted accommodation.

**Figure 1:**
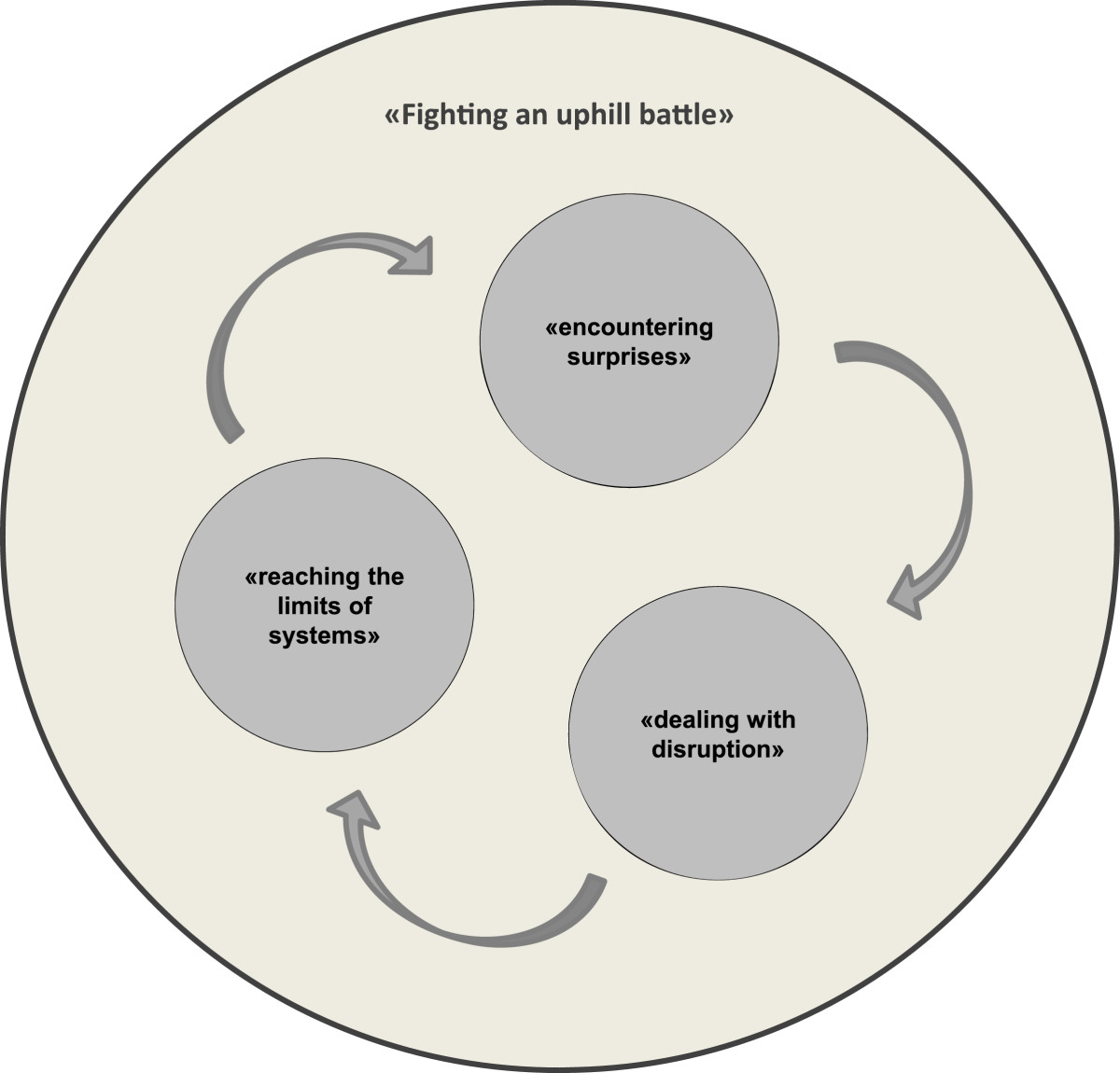
**Constitutive theme and sub-themes.** The constitutive theme “Fighting an uphill battle” fostered by a process of three sub-themes: “encountering surprises”, “dealing with disruption” and “reaching the limits of systems”.

Participants' stories revealed that before starting triple therapy, all believed that, because of the condition of their liver, the recommended treatment was necessary to improve or maintain their health long-term. Perceiving no alternatives, but considering the new triple therapy a “real chance” to overcome the disease, they had chosen to undergo therapy. This decision was strengthened by the knowledge that treatment time would be limited. Prior to the treatment trial's commencement, all had rated it as worth a try. One woman, for example, compared the trial to her HIV therapy:

*I also think it’s good that it only lasts a year and not like in HIV. For that, you have to take it forever. I’ll push it through for a year, even if it’s not pleasant, as long as it does some good. If it doesn’t do any good, then it’s logical that I wouldn’t. (P10, 52 yrs)*Despite their high initial levels of motivation, all test patients later found themselves struggling to continue with the therapy, which was physically, emotionally and socially draining, and which dominated their everyday lives. The constitutive theme characterizing this experience is “Fighting an uphill battle”. This common experience was fostered by a process described by three sub-themes: “encountering surprises”, “dealing with disruption” and “reaching the limits of systems” (see Figure 1).

### Fighting an uphill battle

All patients remembered the treatment period as a huge physical and emotional burden that disrupted their everyday life and pushed them to the limits of their endurance. One man described his experience as follows:

*It’s a quite hard therapy and required a lot of strength. I feel literally how it tears me in all directions. There are phases in which it is easier, but a general feeling is always present. There are up- and downturns inside me like on a roller coaster and sometimes I say it is enough, I cannot stand it any longer, life is too exhausting. (P12, 55 yrs)*

During this trying time, patients were additionally confronted with the limits of the support available from healthcare professionals, family and friends. Amplified by health-threatening and even life-threatening experiences, patients described everyday life as defined by existential battles against innumerable side effects, which started for some immediately and for others later in the course of treatment. In analogy to their experience they used a battle language. For example, patients described that they fought against symptoms or experienced “hell-fire”. In addition to physical and emotional burdens, patients experienced social limitations, even to the point of isolation. One woman (P7, 52 yrs) said she felt half dead during therapy – a “zombie” with no influence on anything and no idea what was happening to her. The symptoms affected many very strongly. For example, they “hit rock bottom”, were “broken on the wheel”, “run over by a truck”, “put through the mill”, or “forced to crawl on all fours”. And the effects could come on very quickly: one had to be admitted to an emergency room “out of the blue”. Those who suffered the most pronounced symptoms agreed that simply maintaining one's course demanded tremendous strength and discipline. Against this stream of suffering, only the hope of a cure drove them onward. One man said:

*And then I battled the rash and told myself that I would carry on, heaven knows how, but I simply would not fail. (P3, 39 yrs)*

The ever-present feeling of “fighting an uphill battle” which beset the patients was amplified by the fact that, despite the information they had received, their symptoms surpassed their imagination.

### “Encountering surprises”

The patients reported that prior to the beginning of therapy, HCV healthcare providers had informed them of side effects they might perceive to varying degrees. Therefore, all expected unpleasant and undesirable symptoms. Based on experience with other medications, including dual therapy, some expected similar symptoms, while others expected greater difficulties from the start. For most, however, the symptoms surpassed all expectations and were unexpected despite preparation. One woman described this as follows:

*Actually, I worked the whole time during the first therapy, yet I can’t remember exactly, but it was never, ever in the way, never to the extent [of the triple therapy side effects], not the icy cold and the drying out, not all that hair loss, either. That’s why I was shocked. (P1, 60 yrs)*

Often the patients lacked the specific vocabulary to denote their symptoms, reporting that they would be difficult to explain to anyone who had never experienced them. Instead, they used vivid descriptions, such as “freezing torpor”, ”frozen skeleton”, “looking like a spotty monster”, “moving like a robot”, wanting to “get the carving knife and scrape that stuff off”, “having the feeling of consisting only of tablets”. Their healthcare providers seemed not to have sufficient experience with other patients to help with finding language. For several participants the symptoms were often so numerous that the burden of suffering could barely be borne. One man described it as follows:

*Life at the moment is only the fight against side effects. Hardly over the worst of one, and up comes the next already. There were maybe four or five most difficult things which were really stressful, and then there were another 20, 30 minor things, like mental problems, difficulties going out among people, suddenly being easily frightened and uncertain. I’m not otherwise a person who lacks self-confidence. Yes, there are a lot of such things. (P2, 39 yrs)*

Even patients with fewer side effects said many of them were agonizing. Also, because symptoms often arose with great speed and force, and the patients lacked any related experiences, they had few strategies for dealing with them. One woman said:

*The side effects were very severe, in particular depression was heavy. What was worst was that it started right away, that everywhere there is mucosa hurt, everything was inflamed. Like the nose, mouth, vaginal area, everywhere. And I also got hemorrhoids right away, within three weeks, and that was brutal pain, I didn‘t know how to deal with it. (P7, 52 yrs)*

The onset of side effects was usually unpredictable. Several might occur suddenly and without warning, or they might develop gradually; so it was impossible to know what condition one would be in even two hours in the future. One man (P5, 48 yrs) said that “in addition to the work, it was quite hard, since there was no central thread and one couldn’t say how one would feel; there was no rhythm”.

The symptoms led to decisive changes in the patients’ lives, against which they struggled. Their daily lives changed totally.

### “Dealing with disruption”

The patients reported that, since starting therapy, nothing was the same as before. Mind and body, and emotional life no longer functioned as usual and considerably reduced the quality of life. One man described the effect as follows:

*Absolutely nothing is the same as it was, neither sleeping nor feeling, simply mind or body, it’s not the same any more, no; even when I touch myself, the whole feeling is simply different; smelling oneself changed; there’s a disgusting taste in the mouth and food tastes different. Nothing, really nothing is the same anymore. (P2, 39 yrs)*

One serious problem for the patients was their lack of productivity, which was usually attributed to great fatigue and accompanied by an absolute lack of energy and strength. They were unable to satisfactorily perform everyday tasks at home, at work, as partners or as parents, which could result in further difficulties. One woman *(P7, 52 yrs)* said she “could no longer lead a normal life and only slept after work”. In addition, for example, one was “no longer able to open a bottle, concentrate for any length of time at work, or stand the lively children and not react with hostility”. What strength remained was often focused on existential priorities such as one's employment or essential everyday tasks; others were pushed back. A man reported:

*And as for my wife, she doesn’t get what she would like. I can’t give her what I don’t have at the moment. That also has to do with potency, there’s such a loss of strength, loss of desire, it’s really terrible for our relationship because I have no strength. And sexuality is one area in which strength plays an important role, and if it’s not there, there’s frustration. (P12, 55 yrs)*

Prior to therapy, most participants' HCV infections had produced no readily apparent symptoms. Considering their therapy a very personal matter, they discussed it with few others, and hoped to prevent the side effects from becoming obvious. However, these were virtually impossible to conceal, and many were ashamed of their condition and appearance. To protect themselves and their families from gossip and isolation, most carefully selected to whom and how fully they revealed the details of their HCV infection. Otherwise, they developed strategies to avoid disclosure. For example, many withdrew from their social environments during the therapy period, leading some to feel very isolated. One man described this as follows:

*I hid it for one reason because I really didn’t know how people would react. In my environment, it’s a touchy subject and people are talking about who has the disease and why. Another main reason is my ego, I don’t want somebody to judge me as sick. It would be enough to see just one glance from someone that tells me, you think I’m sick. If I’m sick, then I’m sick, I’m absolutely not ashamed of it, but it is doubtlessly a peculiar situation, in which [as long as] I don’t admit that there’s something wrong, there’s nothing wrong. Moreover, if anyone points at me and says "sick", it would be awful. There are, of course, people I could tell this to, surely, but in the background I always think some day he will tell it to another person, that’s why I just keep my mouth closed; I simply didn’t want anyone to know. (P3, 39 yrs)*

The changes which were experienced as decisive were often hard for the patients to bear and they needed a lot of strength and willpower to battle on. This also brought them to the limits of their resilience.

### “Reaching the limits of systems”

Initially the patients were confident that they could complete their therapy regardless of the difficulty. However, the changes they recalled as most decisive pushed them to the limits of their strength, willpower and resilience. Struggling with symptoms, they described losing their feeling of self, which left them unable to assess themselves appropriately. This sometimes led to dangerous situations, in which they felt very dizzy or even collapsed. Some recalled how limited their judgments and actions became, or how they feared losing control because of depression or aggression. One man explained:

*There were situations in which I felt so awful that I almost wanted nothing so much as to be admitted to hospital,…at the end of my rope, emotionally and physically. I know when I’m sick, that usually it will pass, and I don’t see the doctor. But this time the limit was overstepped where I am no longer able to judge, and that was a certain danger, since I live alone. There were situations in which…I would have best liked to be in hospital [outpatient clinic] that I was judged by someone else. (P2, 39 yrs)*

Most patients regarded their HCV healthcare providers as valuable sources of support regarding unpredictable symptoms. Offering short-notice appointments via telephone, text message or email, these low-profile, reliably accessible clinicians were extremely helpful to evaluate symptoms or problems with treatment regimens. Concerning symptom control, though, while patients understood that the newness of the protease-inhibitor-based triple therapy made full management impossible, they were surprised at how limited the clinicians' knowledge was. Nevertheless, they appreciated clinicians who empathized with them and assisted in any way in their battle against side effects. This gave them a feeling of support and partnership in seeing their therapy through:

*I had the feeling that even for my nurse and doc this was not routine. Then I realized they were fighting along with me. I really absolutely knew: I suffer from something they don’t really know about. But let’s say, at the worst point, both of them stood with me. (P3, 39 yrs)*

Patients expected healthcare providers to accurately evaluate their health status and take the lead when they themselves were too impaired to assess or react appropriately to a crisis. One woman (P4, 63 yrs) recalled being unaware that she was approaching the point of collapse, and emphasized that, though she understood her clinicians' lack of specific experience, it would have been helpful if one had intervened and sent her to hospital earlier.

Many participants recounted how their inability to describe their symptoms had sometimes prevented them from informing their care teams either promptly or clearly. Also, due to their high number of symptoms, they usually mentioned only those that caused the most stress. Further, they were unaccustomed to reporting symptoms emphatically enough for them to be noted. In retrospect, patients rated open communication with clinicians, including discussions of even the slightest change during therapy, as essential. One noted that the care personnel could not be expected to be “clairvoyant”.

Patients had tremendous faith in their clinicians' advice. Despite information about side effects, and even in difficult situations, many followed therapy instructions precisely and without question. One man reported:

*I simply took the medication without question and thought, you’ve got to take this every eight hours; but I didn’t think anything bad, I simply took it without question. The doctor said I must take it this way. At the end it was difficult, but I didn’t know who was to blame, then, when I suddenly had attacks of shivering. I never would have thought it was because of the medication, someone should have told me more clearly. (P9, 61 yrs)*

Beyond their clinical care, several patients explained that, considering their often very poor physical and emotional condition, they genuinely needed and received extensive support in everyday tasks, and said support from partners, family and friends helped them endure the therapy. These people helped with such tasks as housework or organizing and keeping appointments, and even occasionally supplied motivational talks or instructions for action. This level of assistance usually entailed a great challenge for the supporting person, and required close cooperation with health care providers. Conversely, living alone without support during the therapy period was unanimously rated as problematic. One man *(P6, 40 yrs)* said “I would have missed all my appointments because of drowsiness if my father hadn’t helped me with the appointments and driven me there”.

The majority of patients found medication management difficult and time-consuming. However, even though their regimens required injections, timely intake of medications three times a day and food restrictions, none mentioned this explicitly until asked. Once the topic had been introduced, though, all acknowledged the importance of taking medications exactly on time. They also described phases during which taking medication was easier and others when they had to force themselves to overcome feelings of aversion. Nonetheless, despite the difficulties, to maximize their chances of attaining a cure, they summoned the necessary discipline, and eventually taking medication became routine. Of the entire participant group, only two men, both formerly IDUs from substance abuse treatment clinics, reported that they couldn’t take the medication three times daily as prescribed by the doctor because they were too drowsy and couldn’t wake up on time. Their IFN injections were administered to them weekly by a nurse, which both rated positively. One was able to reduce his medication intake to twice daily after consultation with his health care providers, enabling regular administration from then on. The other terminated PI intake due to unbearable side effects and continued with dual therapy.

In summary, all patients considered the triple therapy an enormous physical and emotional battle: perseverance demanded all of their willpower and discipline. Their motivation was the possibility of cure and adherence with their medication regimen was not a foremost topic, in spite of the radical changes and burdens brought on by the many side effects. These were often unexpected and difficult to discuss or manage. As a result, patients operated at the very limits of their endurance, and all collaboration between HCPs and patients was exploratory, i.e., as PI therapy was extremely new, the clinicians' experience was severely limited and related literature remained scant. Still, regardless of their potential therapeutic success, all of the patients were confident of the value of their decision to undergo therapy.

## Discussion

This thematic analysis provides insights into HCV patients' experiences with PI based triple therapy. Before beginning their therapy, these patients were powerfully motivated to improve their health and achieve a cure. For dual therapy, patients have associated concerns regarding disease progression, loss of liver function and other potential health problems with treatment initiation [43, 44]. However, this study group's homogeneously high motivation might not reflect the attitude of the majority of HCV patients in need for treatment. Studies have shown that large proportions of patients (10% - 44%) undergoing dual therapies typically discontinue treatment [15, 43, 45]. Although we included participants from three different outpatient clinics and used maximum variation sampling to ensure diverse characteristics and living circumstances, there might have been an earlier clinical selection of highly motivated patients treated with the then-newly-available triple therapies and the next generation of interferon-free treatments.

Soon after starting triple therapy, our highly-motivated study group found themselves struggling. The constitutive theme–“Fighting an uphill battle”–describes the patients' common existential experience of coping with the therapy's side effects and indirect negative consequences. Bell et al. describe similar existential treatment experiences among cancer patients receiving adjuvant chemotherapy [46]. However, in contrast to cancer patients, who saw their symptoms as a means of tracking treatment effectiveness and increasing the possibility of remission, this study's HCV patients indicated beliefs congruent with research on triple therapy, i.e., that it numerous side effects, including possible life threatening adverse events, are to be expected, but that no associations exist between symptoms and success [18, 47].

In this study, despite information from healthcare providers and research oriented treatment reports, the symptoms experienced surpassed the patients' expectations (as described in the “Encountering surprises” subtheme). Across chronic conditions, high symptom numbers, high symptom distress and low perceived symptom manageability have negative impacts on health outcomes including quality of life and medication adherence [48, 49]. Given the high number of HCV patients who commonly interrupt dual therapy and the high symptom burden of triple therapy, it can be hypothesized that mitigating patient distress via early symptom management support will increase successful completion of therapy and would decrease dropout rates in less persistent patient groups. And while even healthcare providers were occasionally surprised by the severity and diversity of the new treatment's side effects, the first step of effective symptom management is to assess patients' perceptions of their symptoms [50]. However, our records illustrate very clearly that the participants' symptom experiences were often so foreign to them that only metaphoric language was sufficient to explain them. Therefore, standard biomedical symptom assessment by health care providers might not be sufficient to capture the details of symptoms or symptom clusters that impact safety, adherence and treatment persistence. And with the prospect of HCV treatment options appearing in the near future, a proactive and patient-oriented system of symptom assessment will remain an important component of clinical HCV management. On the one hand these treatments are expected to entail fewer and less severe adverse events; on the other, they will also be used in patients in advanced stages of the disease, suffering co-morbidities and living in more complex circumstances.

As described above in the subtheme “Reaching the limits of systems” the therapy's drastic physical and emotional effects fundamentally disrupted participants’ everyday lives and severely limited their social interactions. Similar phenomena have been described for symptomatic but untreated HCV patients, as well as for other chronic conditions [51].

However, the radical social changes described by our study participants appeared heavily influenced by their fear of stigmatization. In order to protect themselves from negative social consequences, it is common for patients with stigmatized infectious diseases such as HIV and HCV to make careful disclosure decisions and in some cases conceal their conditions as fully as possible [52–54]. Our illness-experienced study group described a range of selective disclosure management strategies that had apparently been effective before the start of their triple therapy. During treatment, though, the sudden appearance of visible symptoms led to isolation when support was most needed. In some cases, though, the perception of societal rejection might simply have been internalized, i.e., the fear of stigmatization may have led some to project it onto those around them. Recent evidence shows promising approaches to combatting internalized stigma (self-stigmatization) by improving both self-esteem and help-seeking behavior [55, 56]. To minimize stress and improve social support during HCV treatment, healthcare providers should discuss stigmatization fears and disclosure management strategies during consultations both before and during treatment.

The importance of social support is also apparent in the “Reaching the limits of systems” subtheme. Participants recalled both how their symptoms sometimes impaired their judgment and how, during these periods of diminished capacity, the support of family members and close friends was crucial. The positive effects of social support on chronically ill patients' health outcomes is well documented. For example, for patients living with HIV and receiving antiretroviral treatment, a stable partnership is associated with a slower progression to AIDS or death [57]; and among patients living with HCV, those who are married are more likely to complete dual therapy [43]. In addition to companionship, close social contact is known to have multifaceted benefits, including emotional, instrumental, informational support [34, 58, 59].

Since study patients revealed problems with judgment and efficacy concerning sometimes life threatening symptoms, a sound knowledge of HCV treatment and decision making skills is important not only for patients but for their close support persons. This requires healthcare providers to identify close support persons and integrate them into patient education programs. Where social contact with support persons is limited, professional support, e.g., community care, should be intensified.

Based on current evidence and clinical expertise, difficulties in adherence with the complex triple therapy regimens are to be expected [18, 28, 59]. Surprisingly, this study's patients did not raise the issue of medication management. However, as some patients mentioned difficulties when asked, adherence issues may simply have been eclipsed by the intensity of the medications' side effects. Given that heavy symptom burdens (often much lighter than those described by this study's patients) are known to impact adherence, and that poor adherence to dual therapy is known to impact virological response [25–27], it is important to integrate non-judgmental adherence assessment into routine clinical care.

The insights provided by this study illustrate the need to assess patient perspectives of new treatment options in ‘real-life’ settings–as opposed to those of clinical trials. In clinical care, clinicians routinely treat patients who would have been excluded from clinical trials due to medical, social or behavioral problems. Research shows that exactly such medical or socio-behavioral challenges impair results in patients with HCV [42, 60, 61]. Therefore, despite the availability of new HCV treatment options, it remains important to assess patients’ treatment experiences with various other regimens where, based on information from clinical trials, these treatments offer higher tolerability and manageability. Furthermore, considering the extremely high cost of the currently-available IFN-free regimes with improved tolerability, it can be expected that they will not be used in all countries.

### Limitations

The study was limited in two ways: a) its selective recruitment and b) its single-point-in-time interviews. Recruitment was influenced by the fact that participants would be among the first in Switzerland to be treated with the then-newly available protease inhibitors and were therefore both unusually motivated and closely monitored. Single-point-in-time interviews might not have shown individual processes in the treatment experience as well as serial interviews over the course of treatment. However, while the study's qualitative design may limited the results' applicability to other groups of HCV patients, it allowed deep insights into topics such as the participants' inability to describe their symptoms, which could not have been investigated using quantitative assessments.

## Conclusions

For patients undergoing HCV triple therapy, the burden is extreme, but can be lightened by early and continuous support with the negative effects of social stigma and self-stigmatization. During treatment, social support can be strengthened by the integration of close support persons into patient education. An important goal of this process is to build and strengthen collaborative relationships between patients, support persons, and healthcare providers concerning clinical decision-making. In this respect, wherever possible, health providers should eliminate access barriers and use patient-centered communication styles to assess and support symptom management and medication adherence. With the introduction of DAAs to ‘real-life’ settings, high accessibility and patient-centered symptom and adherence assessment will demand multidisciplinary team approaches that integrate input from both the patients and their close support persons [53, 55, 56].

## Electronic supplementary material

Additional file 1: Table S1: The six phases of thematic analysis by Braun and Clarke [38]. (PDF 108 KB)

Additional file 2: Table S2: 15 Item Checklist by Braun and Clarke [38]. (PDF 123 KB)

Below are the links to the authors’ original submitted files for images.

Authors’ original file for figure 1
