# Supplementary material for: “Fighting an uphill battle”: experience with the HCV triple therapy: a qualitative thematic analysis
Source: BMC Infect Dis. 2014 Sep 18;14:507. doi: 10.1186/1471-2334-14-507 (PMC4174651; doi:10.1186/1471-2334-14-507)
Supplement: Supplementary file 1 — Additional file 1: Table S1: The six phases of thematic analysis by Braun and Clarke [38]. (PDF 108 KB) [file 12879_2014_3826_MOESM1_ESM.pdf]

Additional file 1 The six phases of thematic analysis by Braun and Clarke (2006)

Table 1: Phases of Thematic Analysis

Phase	Description of the process
1. Familiarising yourself with your data:	Transcribing data (if necessary), reading and re-reading the data, noting down initial ideas.
2. Generating initial codes:	Coding interesting features of the data in a systematic fashion across the entire data set, collating data relevant to each code.
3. Searching for themes:	Collating codes into potential themes, gathering all data relevant to each potential theme.
4. Reviewing themes:	Checking in the themes work in relation to the coded extracts (Level 1) and the entire data set (Level 2), generating a thematic 'map' of the analysis.
5. Defining and naming themes:	Ongoing analysis to refine the specifics of each theme, and the overall story the analysis tells; generating clear definitions and names for each theme.
6. Producing the report:	The final opportunity for analysis. Selection of vivid, compelling extract examples, final analysis of selected extracts, relating back of the analysis to the research question and literature, producing a scholarly report of the analysis.
