# Supplementary material for: “Fighting an uphill battle”: experience with the HCV triple therapy: a qualitative thematic analysis
Source: BMC Infect Dis. 2014 Sep 18;14:507. doi: 10.1186/1471-2334-14-507 (PMC4174651; doi:10.1186/1471-2334-14-507)
Supplement: Supplementary file 2 — Additional file 2: Table S2: 15 Item Checklist by Braun and Clarke [38]. (PDF 123 KB) [file 12879_2014_3826_MOESM2_ESM.pdf]

Additional file 2 15 Item Checklist by Braun and Clarke (2006)

Table 2: A 15-Point Checklist of Criteria for Good Thematic Analysis

Process	No.	Criteria
Transcription	1	The data have been transcribed to an appropriate level of detail, and the transcripts have been checked against the tapes for 'accuracy'.
Coding	2	Each data item has been given equal attention in the coding process.
	3	Themes have not been generated from a few vivid examples (an anecdotal approach), but instead the coding process has been thorough, inclusive and comprehensive.
	4	All relevant extracts for all each theme have been collated.
	5	Themes have been checked against each other and back to the original data set.
	6	Themes are internally coherent, consistent, and distinctive.
Analysis	7	Data have been analysed - interpreted, made sense of - rather than just paraphrased or described.
	8	Analysis and data match each other - the extracts illustrate the analytic claims.
	9	Analysis tells a convincing and well-organised story about the data and topic.
	10	A good balance between analytic narrative and illustrative extracts is provided.
Overall	11	Enough time has been allocated to complete all phases of the analysis adequately, without rushing a phase or giving it a once-over-lightly.
Written report	12	The assumptions about, and specific approach to, thematic analysis are clearly explicated.
	13	There is a good fit between what you claim you do, and what you show you have done - i.e., described method and reported analysis are consistent.
	14	The language and concepts used in the report are consistent with the epistemological position of the analysis.
	15	The researcher is positioned as *active* in the research process; themes do not just 'emerge'.
