# Supplementary material for: “Fighting an uphill battle”: experience with the HCV triple therapy: a qualitative thematic analysis
Source: BMC Infect Dis. 2014 Sep 18;14:507. doi: 10.1186/1471-2334-14-507 (PMC4174651; doi:10.1186/1471-2334-14-507)
Supplement: Supplementary file 3 — Authors’ original file for figure 1 [file 12879_2014_3826_MOESM3_ESM.pdf]

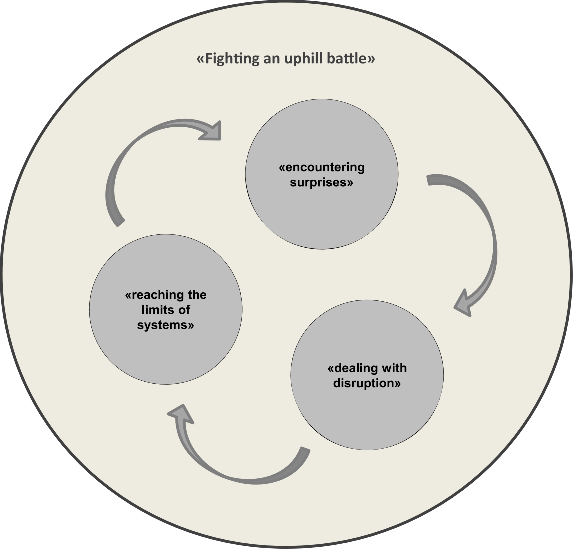
«Fighting an uphill battle»
«encountering surprises»
«dealing with disruption»
«reaching the limits of systems»
